# Supplementary material for: Duration of Neonatal Antibiotic Exposure in Preterm Infants in Association with Health and Developmental Outcomes in Early Childhood
Source: Antibiotics (Basel). 2023 May 26;12(6):967. doi: 10.3390/antibiotics12060967 (PMC10295560; doi:10.3390/antibiotics12060967)
Supplement: Supplementary file 1 [file antibiotics-12-00967-s001.zip › antibiotics-2390152-supplementary.pdf]

Supplementary Tables

Supplementary Table S1. Definition of bronchial obstructive disease, allergic rhinitis, atopic dermatitis, food allergy and recurrent respiratory tract infections

Outcome	Age category	Definition based on questions in the Electronic Portal
Bronchial obstructive disease	2-7 years	Both questions have been answered with 'yes'<br>1. Has your child had wheezing or whistling in the chest at any time in the last 12 months?*)* [1]<br>2. Does your child use puffs/nebulization of either or both<br>1. a short-acting agent to widen the airway (eg Ventolin®, Salbutamol, Bricanyl®)?<br>2. maintenance medication? (These are puffs that your child should use every day, for example: Pulmicort®, Flixotide®, Seretide®, Qvar®…)
Moderate to severe bronchial obstructive disease	2-7 years	*(Based on diagnostic criteria for asthma National Asthma Education and Prevention Program's Expert Panel Report 3, 2007 [2])*<br><br>Either 1 or both of the following criteria fulfilled:<br>1. One of the following answers on the question "How often did your child use short-acting puffs/nebulizers in the last month?<br>a. 1-4 times per week;<br>b. On most days: 1 – 2 x per day;<br>c. On most days: 3 – 4 times a day;<br>d. On most days: more than 6 times a day<br>2. The question "Does your child use puffs/nebulization of maintenance medication (These are puffs that your child should use every day, for example: Pulmicort®, Flixotide®, Seretide®, Qvar®…)?" answered with "yes"

Allergic rhinitis	2-7 years	In the past 12 months, did your child experience sneezing, a runny, or a blocked nose, an itchy nose, red or swollen eyes, itchy or burning eyes, tearing up, when he/she did not have a cold/the flu? [1]
Atopic dermatitis	2-7 years	Both questions have been answered with 'yes':<br>1. Has your child ever an itchy rash which was coming and going for at least 6 months? [1]<br><br>2. Has this itchy rash at any time affected any of the following places: the folds of the elbows, behind the knees, in front of the ankles, under the buttocks, or around the neck, ears or eyes? [1]
Suspected food allergy/intolerance	2-7 years	Both questions have been answered with 'yes' (*adapted from Kansen et al. [3]*):<br>1. Does your child have a food allergy or do you think your child has a food allergy?<br>2. Did you/your child have one or more of the following symptoms?<br><br>- Hives<br>- Oral allergy (tingling in mouth or throat)<br>- Angio-oedema (swelling of mouth/tongue/lips and/or eyes)<br>- Lower respiratory symptoms (difficulty with breathing, squeaky respiration and/or shortness of breath)<br>- Upper respiratory symptoms (swelling of throat, soreness, coughing, running nose, blocked nose, sneezing, itchy/red/tearing eyes, feeling of swollen throat)<br>- Vomiting OR stomach aches OR nausea<br>- Drowsiness OR loss of consciousness
Suspected food allergy	2-7 years	Suspected food allergy/intolerance (cfr. supra) excluding children who were reported to only have abdominal symptoms after ingestion of cow's milk
Proven food allergy by health care provider	2-7 years	Question 1 has been answered with "yes", question 2 has been answered with "the result was positive"<br>1. Has your child ever been tested for food allergies?<br>2. What was the result of the test?
Anti-allergic treatment	2-7 years	Free text answer for: "Does your child have any anti-allergic medicine at home? Which ones?"

		Potential answers: desloratadine, cetirizine, epipen
Surgery due to recurrent respiratory infection	2-7 years	The following question has been answered with one or more of the listed options: “Has your child ever undergone surgery for recurrent ear or throat infections?<br>- Yes, my child got ear tubes<br>- Yes, my child got his/her tonsils removed<br>- Yes, my child got his/her nose polyps removed
Acute lower respiratory tract disease	2-7 years	The following question has been answered with one or more of the listed options: “Has your child ever visited a doctor for the following reasons?”<br>- RSV (RS virus) infection or bronchiolitis<br>- Bronchitis or pneumonia<br>- Acute asthma-like attack (with shortness of breath and wheezing or whistling in the chest)

Supplementary Table S2. Definition of constipation and gastroesophageal reflux

**Outcome**	**Age category**	**Definition based on questions in the Electronic Portal**
ROME IV criteria for functional constipation	2-7 years	At least two of the questions have been answered with the listed options (adapted from Benninga et al., 2016 [4]):<br>1. What does your child's stool usually look like?<br>a. Loose hard droppings, like nuts (hard to evacuate) (Bristol Stool Form Scale (BSS) 1 [5])<br>b. Like a sausage, but lumpy (BSS 2 [5])<br>2. On average, how often does your child go to the toilet for stools?<br>a. 2x per week or less<br>3. If your child is potty/toilet trained, how often does he/she have stool in his/her pants?

		a. 1 or more soiled pair of pants per week<br>4. Does your child hold stools for too long?<br>a. Yes<br>5. Does your child sometimes have so many hard stools that it is difficult to flush<br>a. Yes
Functional constipation	2-7 years	Either 1 or both of the following criteria fulfilled:<br>1. At least two of the ROME IV criteria present (cfr. supra)<br>2. Parent's reported laxative use (e.g. lactulose or macrogol)
Gastroesophageal reflux	2-7 years	Free text answer for "Did your child use antacid medication in the past three months? Which ones?"<br>Potential answers: "ranitidine", "(es)omeprazole"

References Table S1 and S2

1. Asher, M.I., et al., *International Study of Asthma and Allergies in Childhood (ISAAC): rationale and methods.* Eur Respir J, 1995. **8**(3): p. 483-91.
2. *Expert Panel Report 3 (EPR-3): Guidelines for the Diagnosis and Management of Asthma-Summary Report 2007.* J Allergy Clin Immunol, 2007. **120**(5 Suppl): p. S94-138.
3. Kansen, H.M., et al., *Risk factors for atopic diseases and recurrent respiratory tract infections in children.* Pediatr Pulmonol, 2020. **55**(11): p. 3168-3179.
4. Benninga, M.A., et al., *Childhood Functional Gastrointestinal Disorders: Neonate/Toddler.* Gastroenterology, 2016.
5. Lewis, S.J. and K.W. Heaton, *Stool form scale as a useful guide to intestinal transit time.* Scand J Gastroenterol, 1997. **32**(9): p. 920-4.

Supplementary Table S3. Linear and logistic regression analysis for health per 7 days of gestational age, including confounding factors that were used in the multivariate regression analyses

	Missing values out of 347	Mean (SD)/ n (%)	Gestational age (per 7 days) Odds ratio/regression coefficienta [95%CI]	p-value	Confounders in the multivariate analysis
Growth and development					
Weight Z-score at ca. 24 months' CA, mean (SD)	69	-0.47 (1.06)	0.10 [0.02 – 0.18]	0.02*	NABE, birth weight Z-score, mode of delivery
			Adj: 0.10 [0.01 – 0.19]	**0.02***	
Stature Z-score at ca. 24 months' CA, mean (SD)	71	0.14 (1.01)	0.05 [-0.03 – 0.13]	0.18	Birth weight Z-score, mode of delivery
			Adj: 0.08 [0.002 – 0.16]	**0.04***	
BSID-III Cognitive or Motor score <90, n (%)	67	78 (28)	0.85 [0.72 – 1.01]	0.06	NABE, invasive ventilation (<28 days' age)
			Adj: 0.94 [0.78 – 1.13]	0.49	
BSID-III cognitive score, mean (SD)	84	101 (13)	0.43 [-0.58 – 1.44]	0.40	Birth weight Z-score
			Adj: 0.65 [-0.36 – 1.65]	0.21	
BSID-III cognitive score <90, n (%)	42	52 (17)	0.93 [0.77 – 1.12]	0.43	Birth weight Z-score, mode of delivery
			Adj: 0.97 [0.78 – 1.20]	0.75	
BSID-III overall motor score, mean (SD)	134	102 (14)	0.22 [-0.99 – 1.43]	0.72	/
			Adj: NA	NA	
BSID-III overall motor score <90, n (%)	58	63 (22)	0.82 [0.68 – 0.98]	0.03*	NABE, invasive ventilation (<28 days' age)
			Adj: 0.91 [0.74 – 1.11]	0.35	
BSID-III fine motor score <8, n (%)	46	46 (15)	0.83 [0.67 – 1.03]	0.09	/
			Adj: NA	NA	
BSID-III gross motor score <8, n (%)	57	111 (38)	0.85 [0.72 – 1.01]	0.06	NABE
			Adj: 0.91 [0.76 – 1.09]	0.32	

Gastrointestinal symptoms					
Functional constipation, n (%)	18	47 (14)	0.82 [0.68 – 0.98]	0.03*	Invasive ventilation (<28 days' age)
			0.81 [0.66 – 0.99]	**0.04***	
Use of antacid medication, n (%)	7	13 (4)	0.94 [0.66 – 1.34]	0.72	/
			Adj: NA	NA	
Respiratory symptoms					
Sought medical attention due to LRT[a], n (%)	8	114 (34)	0.85 [0.74 – 0.98]	0.02*	Invasive ventilation (<28 days' age)
			0.95 [0.81 – 1.10]	0.49	
Hospitalization due to acute LRT[a] disease[c], n (%)	7	64 (19)	0.92 [0.77 – 1.09]	0.33	/
			Adj: NA	NA	
Ear-nose-throat surgery, n (%)	7	65 (19)	0.99 [0.83 – 1.18]	0.90	/
			Adj: NA	NA	
Wheezing episode in the past 12 months, n (%)	10	88 (26)	1.02 [0.87 – 1.20]	0.79	Invasive ventilation (<28 days' age)
			1.17 [0.98 – 1.39]	0.08	
Bronchodilation past month, n (%)	10	73 (22)	0.99 [0.84 – 1.17]	0.89	Invasive ventilation (<28 days' age)
			Adj: 1.10 [0.92 – 1.32]	0.29	
Bronchodilatation (weekly) , n (%)	15	36 (11)	0.94 [0.75 – 1.17]	0.56	Invasive ventilation (<28 days' age)
			Adj: 1.06 [0.84 – 1.34]	0.64	
Atopic symptoms					
Suspected food allergies , n (%)	20	55 (17)	1.03 [0.86 – 1.25]	0.74	/
			Adj: NA	NA	
Medically confirmed food allergies, n (%)	11	9 (3)	0.83 [0.55 – 1.25]	0.37	/
			Adj: NA	NA	
Atopic dermatitis (ever) , n (%)	7	35 (10)	0.99 [0.79 – 1.24]	0.93	/
			Adj: NA	NA	

Allergic rhinitis or conjunctivitis (past 12 months), n (%)	29	43 (14)	0.94 [0.77 – 1.16]<br>Adj: NA	0.57<br>NA	/
Anti-allergic treatment (current), n (%)	7	6 (2)	0.81 [0.49 – 1.33]<br>Adj: NA	0.41<br>NA	NABE

*p-value<0.05 (bold=adjusted p-value <0.05); aLRT, Lower respiratory tract disease: bronchiolitis, bronchitis, pneumonia, asthma-like attack; Adj, adjusted regression result based on confounding as defined in the method section; BSID-III, Bayley Scale of Infant and Toddler development-III-Dutch version; CA, corrected age; CI, confidence interval; n(%), number and percentage of total number; NA, not applicable; SD, standard deviation

Supplementary Figure

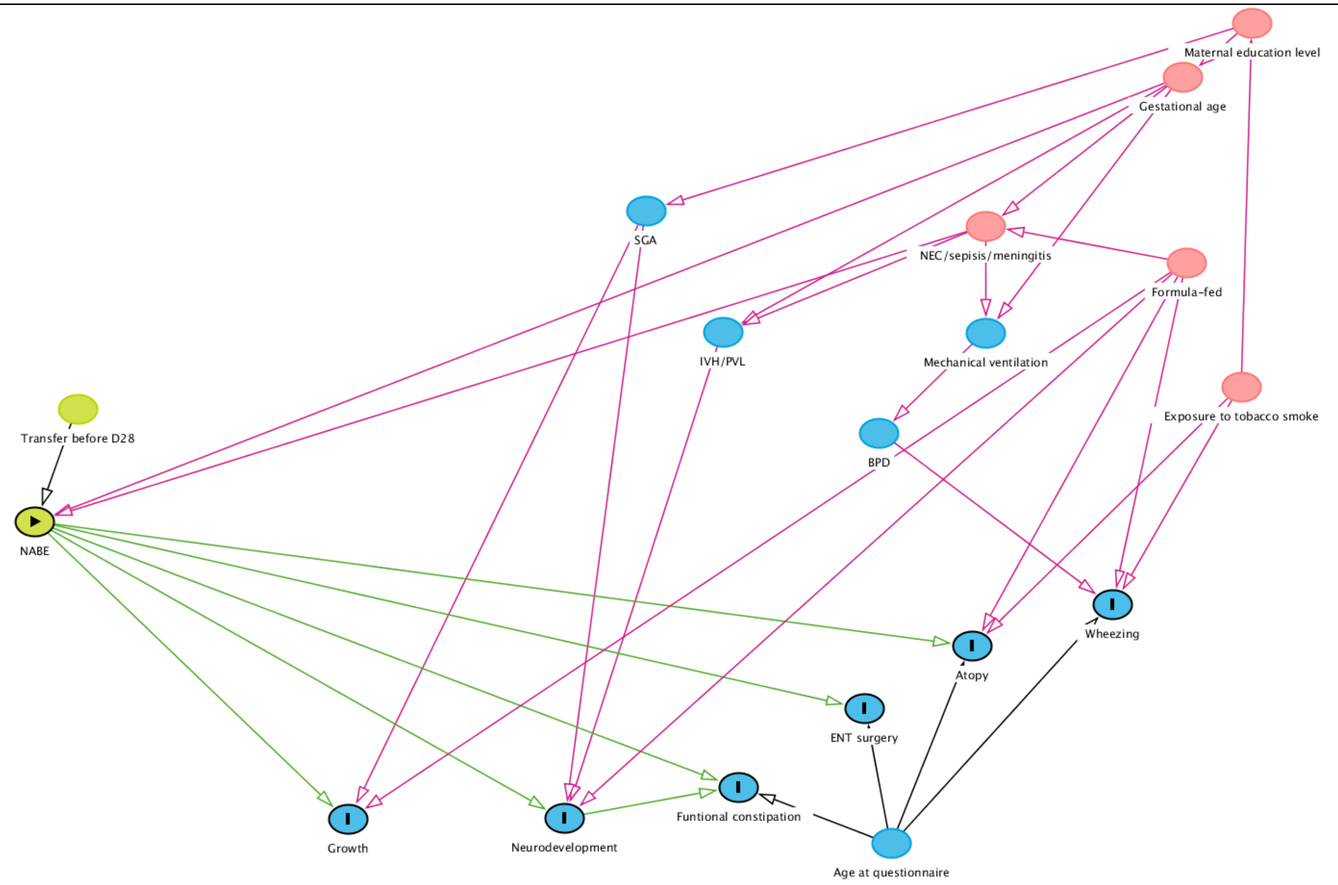

Figure S1. Direct acyclic graph depicting assumed relations between neonatal antibiotic exposure and childhood outcomes.

ENT, ear-nose-throat; IVH, intraventicular hemorrhage, NABE, neonatal antibiotic exposure; PVL, periventricular leukomalacia; SGA small for gestational age
